# A faster way to model neuronal circuitry

**DOI:** 10.7554/eLife.84463

**Published:** 2022-12-02

**Authors:** Andrew P Davison, Shailesh Appukuttan

**Affiliations:** 1 Institut des Neurosciences Paris-Saclay, Université Paris-Saclay, CNRS Saclay France

**Keywords:** computational model, artificial neural net, NMDA, cortex, deep learning, None

## Abstract

Artificial neural networks could pave the way for efficiently simulating large-scale models of neuronal networks in the nervous system.

**Related research article** Oláh VJ, Pedersen NP, Rowan MJM. 2022. Ultrafast simulation of large-scale neocortical microcircuitry with biophysically realistic neurons. *eLife* **11**:e79535. doi: 10.7554/eLife.79535.

Computational modelling and simulation are widely used to help understand the brain. To represent the billions of neurons and trillions of synapses that make up our nervous system, models express electrical and chemical activity mathematically, using equations that they solve with computational methods.

Coarse-grained models of the brain – where each equation represents the collective activity of hundreds of thousands or millions of neurons – have been valuable in helping us understand the coordination of activity across the whole brain (Sanz Leon et al., 2013). The equations from these models can be solved using a normal computer that any researcher might have on their desk. But if we start to investigate how individual neurons and synapses interact to give rise to the collective activity of the brain, the number of equations to be solved becomes enormous. In this case, even powerful supercomputers running flat out for many hours can only simulate the activity of a few cubic millimeters of brain for a few seconds (Billeh et al., 2020; Markram et al., 2015).

Now, in eLife, Viktor Oláh, Nigel Pedersen and Matthew Rowan from the Emory University School of Medicine report on a promising new technique that relies on machine learning tools to greatly accelerate simulations of networks of biologically realistic neurons, without the need for supercomputers (Oláh et al., 2022).

Machine learning approaches have become ubiquitous in recent years, whether it be in self-driving cars, computer-generated art or in the computers that have beat grandmasters in chess and Go. One of the most widely-used tools for machine learning is the artificial neural network, or ANN.

First developed around the middle of the 20th century, ANNs are based on a highly simplified model of how real neurons work (McCulloch and Pitts, 1943; Rosenblatt, 1958). However, it was only in the early 2000s that their use really took off, due to a combination of increased computing power and theoretical advances that allowed ‘deep learning’ (which involves training ANNs with many layers of artificial neurons; reviewed in Schmidhuber, 2015). Each layer in an ANN takes the data from the previous layer as an input, transforms it and feeds it into the next layer, allowing the ANN to perform complex computations (Figure 1).

**Figure 1.**
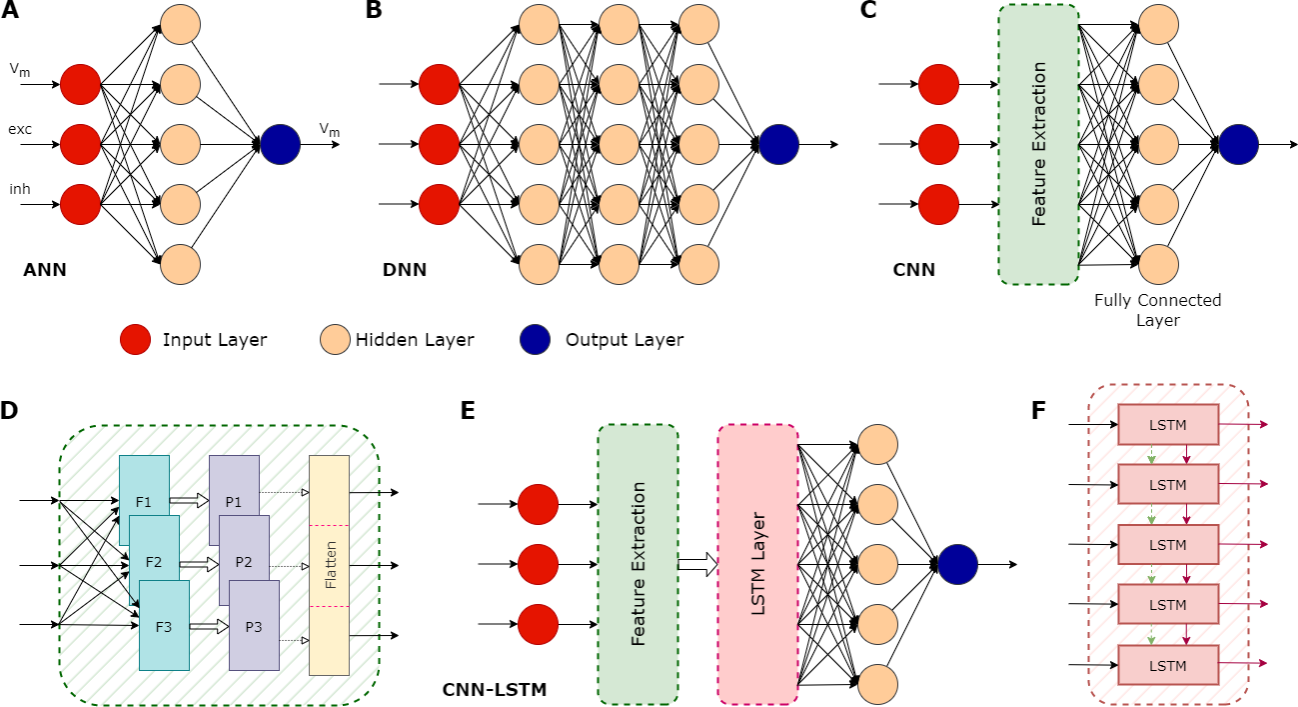
Illustration of various types of artificial neural networks (ANN) and their associated components. (**A**) A basic ANN consists of an input layer (red circles), one or more hidden layers (peach circles), and an output layer (blue circle). In the case of neuronal modelling, the input could be features such as the membrane potential (V_m_), and the excitatory (exc) and inhibitory (inh) synaptic inputs. The hidden layers perform computations on the inputs, with the actual operations depending on the type of ANN. Their objective is to identify features in the inputs and use these to correlate a given input and the correct output. An ANN can have multiple outputs: in this example, the output is a prediction of the membrane potential. (**B**) A deep neural network (DNN) is an ANN with multiple hidden layers. (**C**) A convolutional neural network (CNN) is a type of DNN that can be trained to extract important features contained in the input data, which can then be used as inputs to the other hidden layers, significantly improving the performance of the overall network. (**D**) Some details of the feature extraction process of a CNN, which consists of several hidden layers. First, it has multiple filters (F1, F2, F3), each configured to capture specific features. This process can greatly increase the size of the data, so a pooling layer (P1, P2, P3) is then used to reduce this size. The pooling process does not lead to the loss of valuable data; instead, it helps remove noise and consolidate meaningful data. The flattening layer converts the pooled data into a 1-dimensional stream. This serves as an input for the subsequent fully connected layer, which does the final evaluation to produce the output based on the features extracted by the convolution layers. (**E**) A CNN with a long short-term memory (LSTM) layer. The additional LSTM layer enables the network to benefit from long-term memory, in addition to the existent short-term working memory. (**F**) The LSTM layer achieves this long-term memory through its ability to relay both the cell state (dashed green arrows) and the output generated by each module (solid maroon arrows) across its several modules, allowing the flow of useful information. This enables the network to better identify context in the input data over longer time periods. CNN-LSTMs have been found useful for predicting time series data.

A type of ANN known as a recurrent network has proven to be highly effective at learning to predict changes over time (Hewamalage et al., 2021). In these networks, the activity of a layer of neurons is fed back into itself or into earlier layers, allowing the network to integrate new inputs with its own previous activity. Such ANNs have been used for stock market predictions, machine translation, to accelerate weather and climate change simulations (review in Chantry et al., 2021), and to predict the electrical activity of individual biological neurons (Beniaguev et al., 2021; Wang et al., 2022). Oláh et al. have now developed ANNs that can predict the activity of entire networks of biologically realistic neurons with good levels of accuracy.

First, the team tested several different ANN architectures, and found that a particular type of recurrent neural network – which they call a convolutional neural network with long short-term memory (CNN-LSTM) – was able to accurately predict not only the sub-threshold activity but also the shape and timing of action potentials of neurons. For single neurons, their approach was comparable in speed to traditional simulators. However, when they simulated networks made up of many similar neurons, the performance of the CNN-LSTM was much better, becoming over 10,000 times faster than traditional simulators in certain cases.

In summary, the work of Oláh et al. shows that ANNs are a promising tool for greatly increasing the scope of what can be modelled with generally available computing hardware, reducing the bottleneck of supercomputer availability. Further studies will be needed to better understand the tradeoffs between performance and accuracy for this approach. By clearly describing the successful CNN-LSTM model and providing their source code in a public repository, Oláh et al. have laid a strong foundation for such future exploration.

## References

[bib1] Beniaguev D, Segev I, London M (2021). Single cortical neurons as deep artificial neural networks. Neuron.

[bib2] Billeh YN, Cai B, Gratiy SL, Dai K, Iyer R, Gouwens NW, Abbasi-Asl R, Jia X, Siegle JH, Olsen SR, Koch C, Mihalas S, Arkhipov A (2020). Systematic integration of structural and functional data into multi-scale models of mouse primary visual cortex. Neuron.

[bib3] Chantry M, Christensen H, Dueben P, Palmer T (2021). Opportunities and challenges for machine learning in weather and climate modelling: hard, medium and soft AI. Philosophical Transactions. Series A, Mathematical, Physical, and Engineering Sciences.

[bib4] Hewamalage H, Bergmeir C, Bandara K (2021). Recurrent neural networks for time series forecasting: current status and future directions. International Journal of Forecasting.

[bib5] Markram H, Muller E, Ramaswamy S, Reimann MW, Abdellah M, Sanchez CA, Ailamaki A, Alonso-Nanclares L, Antille N, Arsever S, Kahou GAA, Berger TK, Bilgili A, Buncic N, Chalimourda A, Chindemi G, Courcol JD, Delalondre F, Delattre V, Druckmann S, Dumusc R, Dynes J, Eilemann S, Gal E, Gevaert ME, Ghobril JP, Gidon A, Graham JW, Gupta A, Haenel V, Hay E, Heinis T, Hernando JB, Hines M, Kanari L, Keller D, Kenyon J, Khazen G, Kim Y, King JG, Kisvarday Z, Kumbhar P, Lasserre S, Le Bé JV, Magalhães BRC, Merchán-Pérez A, Meystre J, Morrice BR, Muller J, Muñoz-Céspedes A, Muralidhar S, Muthurasa K, Nachbaur D, Newton TH, Nolte M, Ovcharenko A, Palacios J, Pastor L, Perin R, Ranjan R, Riachi I, Rodríguez JR, Riquelme JL, Rössert C, Sfyrakis K, Shi Y, Shillcock JC, Silberberg G, Silva R, Tauheed F, Telefont M, Toledo-Rodriguez M, Tränkler T, Van Geit W, Díaz JV, Walker R, Wang Y, Zaninetta SM, DeFelipe J, Hill SL, Segev I, Schürmann F (2015). Reconstruction and simulation of neocortical microcircuitry. Cell.

[bib6] McCulloch WS, Pitts W (1943). A logical calculus of the ideas immanent in nervous activity. The Bulletin of Mathematical Biophysics.

[bib7] Oláh VJ, Pedersen NP, Rowan MJM (2022). Ultrafast simulation of large-scale neocortical microcircuitry with biophysically realistic neurons. eLife.

[bib8] Rosenblatt F (1958). The perceptron: a probabilistic model for information storage and organization in the brain. Psychological Review.

[bib9] Sanz Leon P, Knock SA, Woodman MM, Domide L, Mersmann J, McIntosh AR, Jirsa V (2013). The virtual brain: a simulator of primate brain network dynamics. Frontiers in Neuroinformatics.

[bib10] Schmidhuber J (2015). Deep learning. Scholarpedia.

[bib11] Wang T, Wang Y, Shen J, Wang L, Cao L (2022). Predicting spike features of hodgkin-huxley-type neurons with simple artificial neural network. Frontiers in Computational Neuroscience.

